# Breast cancer risk and serum levels of per- and poly-fluoroalkyl substances: a case-control study nested in the California Teachers Study

**DOI:** 10.1186/s12940-018-0426-6

**Published:** 2018-11-27

**Authors:** Susan Hurley, Debbie Goldberg, Miaomiao Wang, June-Soo Park, Myrto Petreas, Leslie Bernstein, Hoda Anton-Culver, David O. Nelson, Peggy Reynolds

**Affiliations:** 1 Cancer Prevention Institute of California, Berkeley, CA USA; 2 Department of Epidemiology and Biostatistics, University of California San Francisco, 2001 Center Street, Suite 700, Berkeley, 94704 California USA; 3 Environmental Chemistry Laboratory, Department of Toxic Substances Control, Berkeley, CA USA; 4 Department of Population Sciences, Beckman Research Institute of the City of Hope, Duarte, CA USA; 5 Department of Epidemiology, School of Medicine, University of California Irvine, Irvine, CA USA; 6 Department of Health Research and Policy, Stanford University School of Medicine, Stanford, CA USA

**Keywords:** Perfluoroalkyl substances, Polyfluoroalkyl substances, PFAS, Breast cancer risk, Case control

## Abstract

**Background:**

Per- and poly- fluoroalkyl substances (PFASs) are a large family of synthetic chemicals, some of which are mammary toxicants and endocrine disruptors. Their potential as breast carcinogens is unclear. Our objective was to evaluate the risk of breast cancer associated with serum PFAS concentrations in a nested case-control study within the California Teachers Study.

**Methods:**

Participants were 902 women with invasive breast cancer (cases) and 858 with no such diagnosis (controls) who provided 10 mL of blood and were interviewed during 2011–2015, an average of 35 months after case diagnosis. PFASs were measured using automated online SPE-HPLC-MS/MS methods. Statistical analyses were restricted to six PFASs with detection frequencies ≥ 95%: PFOA (Perfluorooctanoic acid), PFNA (Perfluorononanoic acid), PFUnDA (Perfluoroundecanoic acid), PFHxS (Perfluorohexane sulfonic acid), PFOS (Perfluorooctane sulfonic acid), and MeFOSAA (2-(N-Methyl-perfluorooctane sulfonamido) acetic acid. Unconditional logistic regression was used to calculate adjusted odds ratios (ORs), estimating the breast cancer risk associated with each PFAS.

**Results:**

For all cases of invasive breast cancer, none of the adjusted ORs were statistically significant but marginally significant ORs < 1.0 were observed for PFUnDA and PFHxS (p-trend = 0.08). Adjusted ORs < 1.0 for PFUnDA and PFHxS were statistically significant (*p* ≤ 0.05) among the 107 cases with hormone-negative tumors but not the 743 with hormone-positive tumors.

**Conclusion:**

Overall, these findings do not provide evidence that serum PFAS levels measured after diagnosis are related to breast cancer risk. The few inverse associations found may be due to chance or may be artifacts of study design. Future studies should incorporate information about genetic susceptibility, endogenous estrogen levels, and measurements of PFASs prior to diagnosis and treatment.

**Electronic supplementary material:**

The online version of this article (10.1186/s12940-018-0426-6) contains supplementary material, which is available to authorized users.

## Background

Per- and poly- fluoroalkyl substances (PFASs) are a large family of synthetic chemicals that have been used in the U.S. since the 1950’s in a variety of industrial applications and consumer products, including non-stick cookware, food packaging, foam fire retardants, water and stain resistant clothing, carpeting and other textiles [1–3]. Characterized by long, highly fluorinated carbon chains that are extremely resistant to biodegradation, the PFASs are among the most persistent and pervasive environmental pollutants [3–7]. Biomonitoring data indicate human exposures are widespread, with the prevalence of detectable levels of some PFAS approaching 100% in many populations [3, 8–22]. Although some of the most well-studied and widely-used PFASs were phased out of use or banned in the U.S. and elsewhere earlier this century, these compounds are still used in many areas of the world and there remain at least 3000 other PFASs currently in use worldwide [2, 3, 6, 23, 24].

Concern for potential cancer risks stems from a large body of laboratory studies that indicate the potential for myriad toxic effects that could play a role in carcinogenesis, including tumor induction, developmental toxicity, hepatoxicity, immunotoxicity, and endocrine disruption [1, 3, 4, 25–32]. Moreover, a number of reviews recently identified PFASs as priority chemicals of specific concern for breast cancer risk [33–35], based on strong evidence from laboratory studies that indicate that some PFASs may act as estrogen disruptors and mammary gland toxicants [1, 30–32].

Cancer risks in humans, however, remain unclear [36, 37]. Of the thousands of PFASs currently in use, only perfluorooctanoic acid (PFOA) has been evaluated for carcinogenicity by the World Health Organization (WHO) International Agency for Research on Cancer (IARC). Citing “limited” evidence for testicular and renal cancer from human and experimental animal studies that is supported by “moderate” evidence for carcinogenic mechanisms, IARC classified PFOA as a “2B possible human carcinogen” [38, 39]. The United States Environmental Protection Agency (US EPA) in its 2005 draft risk assessment initially classified the evidence for the carcinogenicity of PFOA as “suggestive, but not sufficient,” to assess human carcinogenic potential. However, a year later, the US EPA’s Science Advisory Board recommended that US EPA consider reclassifying PFOA’s carcinogenic potential as “likely to be carcinogenic” [40]. US EPA’s final assessment is still forthcoming. Other than PFOA, no other PFASs have been evaluated by health or regulatory agencies to formally classify their carcinogenic potential. A recent review article, however, evaluating the carcinogenic risk of PFOS concluded that the compound could be classified as a group 3 agent “Not classifiable as to its carcinogenicity to humans” in the IARC classification system [36].

Most epidemiologic studies aimed at evaluating cancer risks associated with PFAS exposures have been conducted in occupational cohorts, predominantly comprised of men with insufficient numbers of women to meaningfully examine breast cancer risks [41–48]. Population-based studies are sparse. A pair of analyses from the C8 Science Panel Study conducted in an area of Ohio and West Virginia where the population was known to have long-term high exposures to PFOA due to large-scale environmental releases by a DuPont Teflon manufacturing plant reported statistically significant elevated risks for testicular cancer, as well as suggestively elevated risks for cancers of the kidney, thyroid, and prostate [49, 50]. Despite large numbers of cases, these studies did not detect any risks for breast cancer associated with increases in estimated cumulative PFOA exposure.

To date, only two sets of studies have been designed specifically to evaluate breast cancer risks associated with PFAS exposures. A case-control study of premenopausal women nested within the Danish National Birth Cohort, while reporting a weakly protective effect for perfluorohexane sulfonic acid (PFHxS) and a weakly elevated risk for perfluorooctane sulfonamide (PFOSA), overall found no consistent statistically significant risks for breast cancer [51, 52]. Analyses from a series of small case-control studies among Greenlandic Inuits, however, reported significantly increased risks for a number of individual and summary measures of PFASs, including PFOSA [51, 53–55]. As suggested by the authors of the Greenlandic study, their positive findings may be due to unusually high PFASs body burdens among Greenlandic Inuits, as well as greater susceptibility to exposures due to polymorphisms in genes involved in estrogen biosynthesis and metabolism common among Greenlandic Inuits [54]. The sparseness of the epidemiologic data in the context of these provocative findings among the Greenlandic Inuits, underscore the need to evaluate the risk of PFASs in other study populations.

The objective of the current study was to evaluate the risk of breast cancer associated with serum levels of several PFASs among participants of a large case-control study, nested within the California Teachers Study (CTS), a statewide prospective cohort established specifically to study breast cancer.

## Methods

### Study population

The study population was comprised of 902 invasive breast cancer cases and 858 controls drawn from the California Teachers Study (CTS). The CTS, an on-going prospective cohort study initiated in 1995–1996 primarily to study breast cancer, is comprised of 133,479 female California public school professionals. Details of the creation and conduct of the CTS are described elsewhere [56]. Briefly, since its establishment in 1995–1996 via responses to a mailed questionnaire, the cohort has been followed annually for cancer diagnosis, death, and change of address. State and national mortality files, as well as reports from relatives, are used to ascertain date and cause of death. Address changes for continued follow-up are obtained by several methods including annual mailings, notifications of moves received from participants, and linkages to nationwide consumer reporting companies and the U.S. Postal Service National Change of Address database. Cancer outcomes are identified through annual linkages with the California Cancer Registry (CCR), a legally mandated statewide population-based cancer reporting system. Case ascertainment for the CCR is estimated to be 99% complete and 99% of breast cancer tumors are pathologically confirmed [57].

### Case and control selection

Cases and controls included in the present analysis were drawn from CTS members who had provided a blood sample and completed an interview-administered questionnaire as part of their participation in a separately-funded breast cancer case control study nested within the CTS cohort. Case selection criteria for the nested CTS case control study included: diagnosis with invasive breast cancer (SEER Site code = 26,000) between January 1, 2006 and August 1, 2014; age less than 80 years at diagnosis; no prior history of invasive or in situ breast cancer at cohort entry; and a continuous resident of California from cohort entry until time of diagnosis. Controls were drawn from a probability sample of at-risk CTS cohort members frequency matched to breast cancer cases by age at baseline (5-year age groups), race/ethnicity and CCR regional cancer registry of residence. Participation rates were approximately 55% and 65% for controls and cases, respectively. Compared to non-participants, participants were slightly older (89% versus 86% aged ≥ 40 years) and more likely to be white (91% versus 86%). Active refusals were the most common reason for non-participation, with 29% of controls and 21% of cases refusing, followed by inability to contact/lack of response (12% for controls and 9% for cases). Approximately 4% of controls and 5% of cases were excluded due to illness or death. Interview data and blood specimens were collected from 913 invasive breast cancer cases and 1270 controls during the course of the nested CTS study (May 2011 to October 2015).

Prior analyses of serum PFAS levels among controls in the nested CTS study indicated that PFAS levels were highest during the early months of serum collection [58] – a time, by happenstance, when blood samples were disproportionately collected among controls compared to cases. Therefore, to minimize the potential for bias due to the declining temporal trends in PFAS levels, we excluded participants who provided a blood sample prior to October 2011 (this cut point was chosen based upon a sensitivity analysis we conducted to determine the time interval during which sample collection date no longer affected the estimated risk ratios for breast cancer (*data not shown*)). Furthermore, samples collected during the last 2 months of the CTS nested case control study were not included in the current analysis due to budgetary and timeline constraints of this separately-funded study. Ultimately the participants in the current analyses consisted of all 902 invasive breast cancer cases and 858 controls in the CTS nested case control study who provided a blood specimen and completed an interview-administered questionnaire at blood draw between October 2011 and August 2015. Blood specimens were collected an average of 35 months after case diagnosis (range of interval between diagnosis date and date of specimen collection = 9 months to 8.5 years).

### Serum collection

Blood was collected, usually in participants’ homes, by licensed phlebotomists into a 10 mL BD® tube (catalog#367985, Becton Dickinson, Franklin Lakes, NJ) with clot activator, double gel for transport, and silicone coated interior, using standard phlebotomy techniques. Prior to field processing, specimens were kept on cool packs for at least 30 minutes. Within hours of collection, phlebotomists spun down the clotted blood samples in the field using portable centrifuges to separate the serum portion. Processed samples were then frozen and stored at − 20 °C for 4–6 weeks until transported either via local courier (on cool-packs) or overnight (on dry-ice via FedEx) to the laboratory for chemical analysis. Samples remained frozen during this transportation process. Upon receipt at the laboratory, specimens were stored at − 20 °C until analysis.

### Serum PFASs measurements

Measurement of PFASs in collected sera was conducted using the method as detailed previously [59]. Briefly, 100 μL of serum was diluted in formic acid and spiked with isotopically labeled internal standards before injection into the automated on-line solid phase extraction method coupled to liquid chromatography and tandem mass spectrometry (Symbiosis ™ Pharma, IChrom Solutions, Plainsboro, NJ, and Sciex 4000 QTrap mass spectrometer, Sciex, Redwood City, CA) for clean-up and analysis. Native and isotopically-labeled PFAS standards were purchased from Wellington Laboratories (Shawnee Mission, KS). Within each batch analysis of 20 actual samples, two in-house spiked calf serum samples and NIST 1958 Standard Reference Material were run in duplicate for quality control. The laboratory is proficient in serum PFAS analysis as demonstrated by successful regular participation in proficiency testing (CDC, AMAP).

### Covariate information

Information on potential covariates was derived from a series of surveys, including a self-administered baseline questionnaire completed in 1995–1996 at the initiation of the CTS, five mailed follow-up surveys, as well as a survey administered by a phlebotomist interviewer at the time of blood draw. Data from the interview administered at blood collection were used to characterize age at blood draw in years (40–49/50–59/60–69/70–79/≥80), menopausal status (Post/Pre and Peri/unknown), age (in years) at menopause (Pre and Peri-menopausal/20–43/44–49/50–51/52–53/54–55/56–65/unknown), years since the onset of menopause (Pre-Peri/unknown-menopausal/0–9/10–19/20–29/30–69/unknown) and hormone therapy (HT) use (never used and post-menopausal/ever used and post-menopausal/Pre and peri-menospausal/unknown). Season of blood collection (Winter = December–February; Spring = March–May; Summer = June–Aug; Fall = September–November) was based on the date that the blood sample was collected, as recorded by the phlebotomist. Data from responses to the 1995–1996 baseline questionnaire were used to characterize: age at CTS enrollment in years (20–80); race/ethnicity (White/Black/Hispanic/Asian/PI/other); body mass index (BMI) (16.0–24.9/25.0–29.9/30.0–53.3/unknown); physical activity in hours/week (< 0.5/0.5–3.9/≥4.0/unknown); alcohol consumption in grams/day (none/< 20/≥20/unknown); pack years of smoking (never smoker/≤10/11–20/21–30/≥31/unknown); family history of breast cancer (in a first degree relative -- no/yes/unknown); age at menarche in years (≤11/12–13/≥14/never/unknown); age (in years) at first full-term pregnancy (no full term pregnancy/≤24/25–29/≥30/unknown); breast feeding history (total months) (never pregnant/pregnant without live birth/0/< 6/6–11/≥12/unknown). Detailed information on HT use (never/past/current estrogen use/current estrogen and progesterone use/progesterone only use/premenopausal/unknown) was also gathered from the baseline questionnaire. A variable for weight change (loss of  >5 pounds/gain of  >5 pounds/no change of  >5 pounds/unknown) was calculated based on difference between self-reported weight on the fifth mailed questionnaire (administered in 2012–2013) and the 1995–1996 baseline questionnaire. Dietary consumption (grams/day) of fat (tertiles/unknown), pork (none/< median/ ≥ median/unknown), and total red meat (tertiles/unknown) were derived from information on dietary factors obtained from responses to a modified version of the Block questionnaire included on the 1995–1996 baseline questionnaire [60, 61].

In addition to these survey-based factors, neighborhood characteristics of socioeconomic status and urbanization were also considered as potential covariates as some of our prior work have suggested they may be related to serum PFAS levels [58]. These were derived by linking residence at blood draw to U.S. census data by the block group of residence. A description of these methods appears elsewhere [62, 63].

This initial set of potential covariates was chosen to include established breast cancer risk factors and factors that our prior analyses had identified as correlates to serum PFAS concentrations in this study population.

### Statistical analysis

All analyses were conducted in SAS Version 9.4. The limit of detection (LOD) was defined as three-times the standard deviation of the blank. Because the LOD varied by batch, for the purpose of summarization, we calculated the average LOD across all batches, weighted by the number of samples in each batch. Samples with PFAS concentrations below the LOD were imputed as LOD/$\sqrt{2}$ [64, 65]. To avoid potential bias from such imputation, six PFASs with detection frequencies (DF) below 95% were excluded from the risk analyses including: PFOSA (Perfluorooctane sulfonamide), PFBS (Perfluorobutane sulfonic acid), EtFOSSA (2-(N-Ethyl-perfluorooctane sulfonamido) acetic acid), PFDA (Perfluorodecanoic acid), PFDoDA (Perfluorododeconic acid), and PFHpA (Perfluoroheptanoic acid) so that the risk analyses were restricted to the following six PFAS with DFs ≥ 95%: PFOA (Perfluorooctanoic acid), PFNA (Perfluorononanoic acid), PFUnDA (Perfluoroundecanoic acid), PFHxS (Perfluorohexane sulfonic acid), PFOS (Perfluorooctane sulfonic acid), MeFOSAA (2-(N-Methyl-perfluorooctane sulfonamido) acetic acid (Table 2).

Prior to conducting the risk analyses, a number of exploratory and descriptive analyses were conducted. Frequency distributions of potential covariates were evaluated and chi-square statistics were calculated to assess the statistical significance of differences in distribution by case status. Spearman rank correlations between PFASs were calculated. As many of the PFAS were highly correlated we considered each PFAS separately in our analyses. Because the PFAS concentrations were highly-skewed, values were log_10_-transformed in all subsequent analyses to enable the application of parametric statistical approaches. Smoothing splines were considered in generalized additive models (using PROC GAM) and evaluated to assess potential non-linearities in the relationship between each PFAS and the log-odds of breast cancer but no evidence of non-linearity was observed.

The risk of breast cancer associated with each PFAS was estimated by unconditional logistic regression, using PROC LOGISTIC to generate odds ratios (OR) and 95% confidence intervals (95% CI). PFAS concentrations were considered alternatively in separate sets of models as continuous variables (expressed as log_10_ [PFAS, ng/ML]) and categorical variables (high/medium/low, based on tertiles of the PFAS concentrations in the controls). All regression models included terms for the matching design variables (age at baseline enrollment, race/ethnicity, and region of residence). Crude ORs were calculated from simple models including only these matching design variables. Adjusted ORs were generated from multivariate models, adjusting for potential confounding factors. Starting with the full set of potential covariates (as described in the previous section), covariates for inclusion in the final multivariable models were selected via a two-step process. First, a backwards elimination approach was used, starting with a model that forced inclusion of the PFAS variable, age, race, and region of residence, and retaining all covariates for which the *p*-value for the Wald chi-square was < 0.05. We then further evaluated potential confounders by adding each of the excluded variables back into the model one at a time and evaluated the effect of the change in the estimated odds ratios for the PFAS. Factors that changed the estimated odds ratio for the PFAS by 10% or more were retained in our final multivariable regression models. While we conducted this process separately for each PFAS, it resulted in the same set of covariates for all PFASs. The final set of covariates appear as footnotes to Table 3.

A number of stratified analyses were conducted to evaluate whether risks may differ within certain subsets of our study population. The subsets of interest were chosen a priori after an extensive literature review. Selected subsets included types of breast cancer that are thought to have distinct etiologies, including: pre/peri-menopausal versus post-menopausal cases and cases with tumors that were hormonally responsive, identified as estrogen or progesterone receptor positive (ER+/PR+) tumors versus non-hormonally responsive tumors that were estrogen receptor negative and progesterone receptor negative (ER-/PR-). Furthermore, because toxicological evidence suggests that the effects of exposure to xenobiotic endocrine disruptors such as PFASs may differ depending on the endogenous hormonal milieu in which they occur [66, 67], we evaluated risks separately for: women who had and had not ever used menopausal HT; nulliparous and parous women; and for women with low, medium and high BMI. Multivariable logistic models were built separately for each of these subset analyses, using the same method described above for covariate selection. Final covariates are listed in the footnotes of Tables 4 and 5.

## Results

Selected characteristics of the study population are detailed in Table 1. Participants were predominantly middle-aged and older non-Hispanic white women – reflecting the characteristics of the CTS cohort from which they were selected. Compared to controls, cases were more likely to report a family history of breast cancer, to smoke cigarettes, have a higher BMI, never had a full-term pregnancy and had a later age at first full-term pregnancy (among parous women), and consume more red meat and pork. Among women who had reached menopause by the time of blood draw, the age at menopause did not differ between cases and controls. Post-menopausal breast cancer cases, however, were slightly more likely than controls to have entered menopause more recently (i.e., within 10 years prior to blood draw). The year and season during which blood draw occurred differed between cases and controls with controls more likely than cases to have had their blood samples collected during the initial few months of the study and during the fall and winter months.

**Table 1:** Characteristics of study participants (*n* = 1760), by case-control status

Characteristic^a^	Case	Control	All	*p*-value^b^
	N (%)	N (%)	N (%)	
All	902 (100)	858 (100)	1760 (100)	
Age at baseline enrollment (years)				0.51
20–39	114 (13)	89 (10)	203 (12)	
40–44	121 (13)	125 (15)	246 (14)	
45–49	204 (23)	186 (22)	390 (22)	
50–54	196 (22)	198 (23)	394 (22)	
55–59	152 (17)	135 (16)	287 (16)	
60–80	115 (13)	125 (15)	240 (14)	
Age at blood draw (years)				0.35
40–49	38 (4)	30 (3)	68 (4)	
50–59	127 (14)	108 (13)	235 (13)	
60–69	362 (40)	369 (43)	731 (42)	
70–79	316 (35)	281 (33)	597 (34)	
≥ 80	59 (7)	70 (8)	129 (7)	
Race/Ethnicity				0.40
White	812 (90)	763 (89)	1575 (89)	
Black	14 (2)	7 (1)	21 (1)	
Hispanic	24 (3)	32 (4)	56 (3)	
Asian/Pacific Islander	29 (3)	32 (4)	61 (3)	
Other	23 (3)	24 (3)	47 (3)	
Region of Residence (CCR region)				0.01
1:Santa Clara Region	82 (9)	83 (10)	165 (9)	
2:Central Region	99 (11)	95 (11)	194 (11)	
3:Sacramento Region	110 (12)	134 (16)	244 (14)	
4:Tri-County Region	65 (7)	62 (7)	127 (7)	
5: Inland Empire Region	42 (5)	25 (3)	67 (4)	
6:North Region	68 (8)	44 (5)	112 (6)	
7:Imperial and San Diego Region	60 (7)	48 (6)	108 (6)	
8:Bay Area Region	118 (13)	99 (12)	217 (12)	
9:Los Angeles County	173 (19)	148 (17)	321 (18)	
10:Orange County	85 (9)	120 (14)	205 (12)	
Smoking pack-years				0.03
Never smoker	580 (64)	573 (67)	1153 (66)	
≤ 10	156 (17)	153 (18)	309 (18)	
11–20	47 (5)	60 (7)	107 (6)	
21–30	45 (5)	23 (3)	68 (4)	
≥ 31	42 (5)	27 (3)	69 (4)	
Unknown	32 (4)	22 (3)	54 (3)	
BMI at baseline (kg/m^2^)				0.01
16.0–24.9	496 (55)	519 (60)	1015 (58)	
25.0–29.9	236 (26)	203 (24)	439 (25)	
30.0–53.3	153 (17)	110 (13)	263 (15)	
Unknown	17 (2)	26 (3)	43 (2)	
Weight change (Q5-baseline questionnaire)				0.08
Lost weight	161 (18)	133 (16)	294 (17)	
No change (+/−5lbs)	201 (22)	235 (27)	436 (25)	
Gained weight	346 (38)	317 (37)	663 (38)	
Unknown	194 (22)	173 (20)	367 (21)	
Alcohol consumption (g/day)				0.82
None	278 (31)	250 (29)	528 (30)	
< 20	514 (57)	507 (59)	1021 (58)	
≥ 20	74 (8)	66 (8)	140 (8)	
Unknown	36 (4)	35 (4)	71 (4)	
Physical activity (hours/week)				0.20
< 0.50	74 (8)	51 (6)	125 (7)	
0.50–3.99	460 (51)	438 (51)	898 (51)	
≥ 4.00	367 (41)	366 (43)	733 (42)	
Unknown	1 (0)	3 (0)	4 (0)	
Family history of breast cancer				0.01
No	728 (81)	737 (86)	1465 (83)	
Yes	136 (15)	96 (11)	232 (13)	
Unknown	38 (4)	25 (3)	63 (4)	
Age at menarche (years)				0.39
≤ 11	227 (25)	189 (22)	416 (24)	
12–13	500 (55)	484 (56)	984 (56)	
≥ 14	166 (18)	174 (20)	340 (19)	
Unknown/Never	9 (1)	11 (1)	20 (1)	
Age at first full-term pregnancy (years)				0.01
No full-term pregnancy	233 (26)	186 (22)	419 (24)	
≤ 24	209 (23)	250 (29)	459 (26)	
25–29	257 (28)	257 (30)	514 (29)	
≥ 30	193 (21)	151 (18)	344 (20)	
Unknown	10 (1)	14 (2)	24 (1)	
Breast feeding history (months)				0.26
Never pregnant	173 (19)	132 (15)	305 (17)	
Pregnancy, but no live birth	58 (6)	53 (6)	111 (6)	
0	117 (13)	125 (15)	242 (14)	
> 0 and < 6	152 (17)	130 (15)	282 (16)	
6–11	121 (13)	132 (15)	253 (14)	
≥ 12	269 (30)	270 (31)	539 (31)	
Unknown	12 (1)	16 (2)	28 (2)	
Menopausal status at time of blood draw				0.10
Pre/Peri-menopausal	43 (5)	59 (7)	102 (6)	
Postmenopausal	859 (95)	798 (93)	1657 (94)	
Unknown	0 (0)	1 (0)	1 (0)	
HT use at blood draw^c^				0.06
Never used and postmenopausal	288 (32)	237 (28)	525 (30)	
Ever used and postmenopausal	571 (63)	561 (65)	1132 (64)	
Pre/Peri-menopausal	43 (5)	59 (7)	102 (6)	
Unknown	0 (0)	1 (0)	1 (0)	
HT use at baseline				0.34
Never used	64 (7)	68 (8)	132 (8)	
Past HT use	29 (3)	37 (4)	66 (4)	
Current estrogen use	130 (14)	143 (17)	273 (16)	
Current estrogen and progesterone use	240 (27)	199 (23)	439 (25)	
Progesterone use only	6 (1)	10 (1)	16 (1)	
Pre-menopausal	399 (44)	367 (43)	766 (44)	
Unknown	34 (4)	34 (4)	68 (4)	
Age at menopause (years)				0.22 ^d^
Pre/peri-menopausal	43 (5)	59 (7)	102 (6)	
20–43	143 (16)	151 (17)	294 (17)	
44–49	187 (21)	139 (16)	326 (19)	
50–51	191 (21)	161 (19)	352 (20)	
52–53	112 (12)	102 (12)	214 (12)	
54–55	111 (12)	119 (14)	230 (13)	
56–65	89 (10)	92 (11)	181 (10)	
Unknown	26 (3)	35 (4)	61 (3)	
Years since onset of menopause				0.01^d^
Pre/peri/unknown menopausal status	43 (5)	60 (7)	103 (6)	
0–9	190 (21)	137 (16)	327 (19)	
10–19	29 (32)	305 (36)	596 (34)	
20–29	244 (27)	196 (23)	440 (25)	
30–69	116 (13)	136 (16)	252 (14)	
Unknown	18 (2)	24 (3)	42 (2)	
Dietary fat				0.55
Low tertile	270 (30)	274 (32)	544 (31)	
Middle tertile	284 (31)	261 (30)	545 (31)	
High tertile	278 (31)	269 (31)	547 (31)	
Unknown	70 (8)	54 (6)	124 (7)	
Pork consumption				0.01
None	352 (39)	374 (44)	726 (41)	
Low (< median)	193 (21)	209 (24)	402 (23)	
High (> median)	287 (32)	221 (26)	508 (29)	
Unknown	70 (8)	54 (6)	124 (7)	
Total red meat consumption				0.03
Low tertile	252 (28)	291 (34)	543 (31)	
Middle tertile	279 (31)	262 (31)	541 (31)	
High tertile	301 (33)	25 (29)	552 (31)	
Unknown	70 (8)	54 (6)	124 (7)	
Neighborhood socioeconomic status				0.78
Lowest quintile	21 (2)	25 (3)	46 (3)	
2nd quintile	69 (8)	63 (7)	132 (8)	
3rd quintile	148 (16)	137 (16)	285 (16)	
4th quintile	260 (29)	225 (26)	485 (28)	
Highest quintile	331 (37)	334 (39)	665 (38)	
Unknown	73 (8)	74 (9)	147 (8)	
Neighborhood urbanization				0.92
Rural/Town	106 (12)	90 (10)	196 (11)	
City	245 (27)	230 (27)	475 (27)	
Suburban	440 (49)	426 (50)	866 (49)	
Metro	38 (4)	38 (4)	76 (4)	
Date of blood draw				< 0.01
2011 Oct-Dec	19 (2)	147 (17)	166 (9)	
2012 Jan-Jun	257 (28)	151 (18)	408 (23)	
2012 Jul-Dec	155 (17)	69 (8)	224 (13)	
2013 Jan-Jun	121 (13)	142 (17)	263 (15)	
2013 Jul-Dec	91 (10)	81 (9)	172 (10)	
2014 Jan-Jun	65 (7)	67 (8)	132 (8)	
2014 Jul-Dec	100 (11)	108 (13)	208 (12)	
2015 Jan-Jun	85 (9)	82 (10)	167 (9)	
2015 Jul-Aug	9 (1)	11 (1)	20 (1)	
Season of blood draw				< 0.01
Winter	212 (24)	300 (35)	512 (29)	
Spring	275 (30)	173 (20)	448 (25)	
Summer	242 (27)	125 (15)	367 (21)	
Fall	173 (19)	260 (30)	433 (25)	

^a^Characteristics assessed at CTS baseline enrollment, unless otherwise noted

^b^*p*-value for the Chi-square, comparing cases to controls

^c^HT use was not characterized for pre- and peri-menopausal women

^d^Among postmenopausal women at blood draw

Serum PFAS levels are summarized in Table 2. Summary statistics are not shown for the six PFASs with DFs less than 95%. Similar to reports from other study populations, concentrations were highest for PFOS, followed by PFOA, and PFHxS. Distributions were generally similar for cases and controls, with Wilcoxon rank sum tests indicating no difference in medians (*p* > 0.05) for all PFASs, with the exception of MeFOSAA for which the median was marginally higher in controls compared to cases (*p* = 0.04).

**Table 2:** Serum Concentration of PFAS (ng/mL) among breast cancer cases and controls

	n	DF	LOD	Mean^b^	Median^b^	Min^b^	Max^b^	*p*-value^c^
PFOA (Perfluorooctanoic acid)								
Cases	902	99.8	0.073	2.744	2.350	0.042	39.100	
Controls	858	100.0	0.077	2.938	2.475	0.096	20.200	0.12
PFNA (Perfluorononanoic acid)								
Cases	871^a^	99.0	0.029	0.987	0.850	0.017	7.310	
Controls	849	98.8	0.032	1.036	0.846	0.017	10.400	0.96
PFUnDA (Perfluoroundecanoic acid)								
Cases	902	94.5	0.016	0.155	0.122	0.007	1.030	
Controls	858	95.5	0.018	0.163	0.128	0.007	1.310	0.22
PFHxS (Perfluorohexane sulfonic acid								
Cases	902	99.8	0.022	2.217	1.515	0.011	40.700	
Controls	858	99.9	0.018	2.242	1.605	0.011	21.800	0.16
PFOS (Perfluorooctane sulfonic acid)								
Cases	902	99.8	0.063	8.021	6.695	0.046	39.400	
Controls	858	99.4	0.078	8.320	6.950	0.046	99.800	0.14
MeFOSAA (2-(N-Methyl-perfluorooctane sulfonamido) acetic acid)								
Cases	902	94.4	0.018	0.301	0.152	0.007	4.000	
Controls	858	94.8	0.019	0.331	0.173	0.006	8.370	0.04
EtFOSSA (2-(N-Ethyl-perfluorooctane sulfonamido) acetic acid)								
Cases	902	68.4	0.013	–	–	–	–	
Controls	858	71.3	0.015	–	–	–	–	–
PFBS (Perfluorobutane sulfonic acid)								
Cases	138^a^	8.0	0.053	–	–	–	–	
Controls	294	17.7	0.043	–	–	–	–	–
PFDA (Perfluorodecanoic acid)								
Cases	902	87.3	0.061	–	–	–	–	
Controls	858	88.7	0.053	–	–	–	–	–
PFDoDA (Perfluorododeconic acid)								
Cases	880^a^	8.6	0.096	–	–	–	–	
Controls	858	7.5	0.099	–	–	–	–	–
PFHpA (Perfluoroheptanoic acid)								
Cases	902	64.3	0.028	–	–	–	–	
Controls	858	62.6	0.030	–	–	–	–	–
PFOSA (Perfluorooctane sulfonamide)								
Cases	672^a^	51.6	0.023	–	–	–	–	
Controls	775	65.2	0.019	–	–	–	–	–

*DF* detection frequency, *LOD* average limit of detection, *Min* minimum, *Max* maximum

^a^ Due to laboratory failure 40 PFNA, 1328 PFBS, 22 PFDoA, and 313 PFOSA sample concentrations were not reported

^b^ Descriptive statistics were not generated for the PFASs excluded from analysis due to DF < 95%

^c^*p*-value from the Wilcoxon rank sum test for differences in medians by case-control status

Statistically significant positive correlations were observed between all the PFASs with Spearman Rank Correlations ranging from 0.21 (for PFHxS and PFUnDA) to 0.63 (for PFOS and PFOA). Correlations between the PFASs were generally similar among cases and controls (*data not shown).*

The risks associated with serum PFAS concentrations for all invasive breast cancers in our full study population are summarized in Table 3. Overall, the crude ORs, while having marginally more narrow confidence intervals, were similar to the adjusted ORs. All adjusted ORs were close to 1.0 and not statistically significant – regardless of whether the PFAS was modeled continuously or categorically. Marginally significant ORs < 1.0 for PFHxS and PFUnDA were observed only when modeled as categorical variables (*p*-value for trend = 0.08) but not when modeled as log-linear continuous variables (*p* = 0.18 and *p* = 0.25, respectively).

**Table 3:** Invasive breast cancer risk associated with serum PFAS concentrations among 1760 study participants

PFAS Serum Concentration	# cases	Crude^a^ OR (95% CI)	*p*-value^b^	Adjusted^c^ OR (95% CI)	*p*-value^b^
PFOA					
Low	331	1.00 (ref)	0.32	1.00 (ref)	0.54
Medium	298	0.904 (0.718, 1.139)		0.901 (0.705, 1.152)	
High	273	0.888 (0.698, 1.130)		0.925 (0.715, 1.197)	
log [PFOA, ng/mL]	902	0.718 (0.499, 1.034)	0.08	0.733 (0.496, 1.081)	0.11
PFNA					
Low	285	1.00 (ref)	0.48	1.00 (ref)	0.79
Medium	297	1.083 (0.855, 1.371)		1.043 (0.808, 1.345)	
High	289	1.091 (0.859, 1.384)		1.037 (0.798, 1.348)	
log [PFNA, ng/mL]	871^d^	0.990 (0.724, 1.354)	0.95	0.880 (0.624, 1.240)	0.46
PFUnDA					
Low	312	1.00 (ref)	0.11	1.00 (ref)	0.08
Medium	335	1.080 (0.859, 1.357)		1.106 (0.866, 1.413)	
High	255	0.817 (0.642, 1.039)		0.786 (0.605, 1.020)	
log [PFUnDA, ng/mL]	902	0.855 (0.668, 1.094)	0.22	0.855 (0.653, 1.118)	0.25
PFHxS					
Low	363	1.00 (ref)	0.02	1.00 (ref)	0.08
Medium	263	0.734 (0.580, 0.930)		0.798 (0.621, 1.025)	
High	276	0.762 (0.598, 0.970)		0.801 (0.619, 1.035)	
log [PFHxS, ng/mL]	902	0.780 (0.583, 1.042)	0.09	0.811 (0.596, 1.104)	0.18
PFOS					
Low	318	1.00 (ref)	0.60	1.00 (ref)	0.41
Medium	297	0.955 (0.757, 1.206)		0.883 (0.691, 1.129)	
High	287	0.938 (0.738, 1.193)		0.898 (0.695, 1.161)	
log [PFOS, ng/mL]	902	1.011 (0.754, 1.356)	0.94	0.934 (0.683, 1.277)	0.67
MeFOSAA					
Low	349	1.00 (ref)	0.07	1.00 (ref)	0.29
Medium	278	0.804 (0.638, 1.014)		0.847 (0.663, 1.083)	
High	275	0.812 (0.644, 1.026)		0.877 (0.682, 1.126)	
log [MeFOSAA, ng/mL]	902	0.873 (0.715, 1.066)	0.18	0.960 (0.774, 1.191)	0.71

*OR* odds ratio, *CI* confidence interval

^a^Crude ORs adjusted for matching design variables of age at baseline enrollment, race/ethnicity, and region of residence

^b^for the categorical analysis, the *p*-values represent a test for linear trend with tertiles of PFAS modeled as a 3-level ordinal variable; for the continuous PFAS term, the *p*-value represents the *p*-value of the Wald-statistic for the β-coefficient for the PFAS modeled as a continuous term

^c^ORs adjusted for age at baseline enrollment, race/ethnicity, region of residence, date of blood draw, date of blood draw^2^, season of blood draw, total smoking pack-years, BMI, family history of breast cancer, age at first full-term pregnancy, menopausal status at blood draw, and pork consumption

^d^ PFNA excludes *n* = 40 with non-reportable serum value, (cases: *n* = 31; controls: *n* = 9)

When stratified by menopausal status, the overall pattern of risk estimates were similar to that observed in the full study population (Table 4). The suggestive inverse associations observed in the full study population for PFHxS and PFUnDA persisted across strata of menopausal status and reached statistical significance for PFUnDA among pre/peri-menopausal women (p-trend = 0.04) and for PFHxS among post-menopausal women (p-trend = 0.05).

**Table 4:** Invasive breast cancer risk associated with PFAS concentrations, by menopausal status^a^

PFAS Serum Concentration	Postmenopausal (*n* = 1657)					Pre- or Peri-menopausal (*n* = 102)				
	# cases	Crude Odds Ratio (OR) ^b^		Adjusted Odds Ratio (OR) ^d^		# cases	Crude Odds Ratio (OR) ^b^		Adjusted Odds Ratio (OR) ^e^	
		OR (95% CI)	*p*-value^c^	OR (95% CI)	*p*-value^c^		OR (95% CI)	*p*-value^c^	OR (95% CI)	*p*-value^c^
PFOA										
Low	306	1.00	0.27	1.00	0.49	25	1.00	0.77	1.00	0.62
Medium	287	0.868 (0.682, 1.103)		0.889 (0.689, 1.147)		11	0.881 (0.318, 2.443)		0.888 (0.239, 3.302)	
High	266	0.870 (0.679, 1.116)		0.912 (0.699, 1.189)		7	0.859 (0.264, 2.796)		0.669 (0.143, 3.119)	
log [PFOA, ng/mL]	859	0.696 (0.475, 1.019)	0.06	0.715 (0.476, 1.073)	0.11	43	0.352 (0.077, 1.613)	0.18	0.177 (0.023, 1.342)	0.09
PFNA										
Low	266	1.00	0.92	1.00	0.71	19	1.00	0.06	1.00	0.06
Medium	286	1.016 (0.795, 1.298)		0.978 (0.751, 1.275)		11	1.373 (0.469, 4.017)		1.373 (0.469, 4.017)	
High	277	1.013 (0.791, 1.297)		0.949 (0.723, 1.247)		12	3.117 (0.975, 9.964)		3.117 (0.975, 9.964)	
log [PFNA, ng/mL]	829^f^	0.941 (0.681, 1.301)	0.71	0.837 (0.587, 1.195)	0.33	42 ^f^	1.360 (0.293, 6.309)	0.69	1.360 (0.293, 6.309)	0.69
PFUnDA										
Low	290	1.00	0.14	1.00	0.08	22	1.00	0.20	1.00	0.04
Medium	318	1.096 (0.863, 1.391)		1.100 (0.853, 1.419)		17	0.847 (0.341, 2.101)		0.476 (0.157, 1.445)	
High	251	0.828 (0.646, 1.062)		0.784 (0.600, 1.024)		4	0.365 (0.090, 1.469)		0.179 (0.033, 0.970)	
log [PFUnDA, ng/mL]	859	0.838 (0.649, 1.082)	0.18	0.827 (0.628, 1.091)	0.18	43	0.769 (0.251, 2.354)	0.65	0.377 (0.095, 1.494)	0.16
PFHxS										
Low	334	1.00	0.01	1.00	0.05	29	1.00	0.92	1.00	0.18
Medium	253	0.700 (0.548, 0.893)		0.762 (0.589, 0.986)		10	1.102 (0.381, 3.192)		0.659 (0.184, 2.359)	
High	272	0.733 (0.573, 0.939)		0.769 (0.592, 0.999)		4	0.817 (0.170, 3.936)		0.286 (0.043, 1.892)	
log [PFHxS, ng/mL]	859	0.728 (0.539, 0.985)	0.04	0.759 (0.511, 1.044)	0.09	43	1.095 (0.317, 3.788)	0.89	0.577 (0.143, 2.325)	0.44
PFOS										
Low	293	1.00	0.35	1.00	0.26	25	1.00	0.23	1.00	0.57
Medium	284	0.903 (0.708, 1.150)		0.843 (0.653, 1.088)		13	1.620 (0.559, 4.691)		1.796 (0.493, 6.546)	
High	282	0.888 (0.694, 1.135)		0.860 (0.661, 1.118)		5	2.345 (0.439, 12.536)		1.208 (0.163, 8.944)	
log [PFOS, ng/mL]	859	0.951 (0.703, 1.288)	0.75	0.885 (0.641, 1.223)	0.46	43	2.006 (0.472, 8.524)	0.35	0.900 (0.166, 4.876)	0.90
MeFOSAA										
Low	329	1.00	0.09	1.00	0.31	20	1.00	0.99	1.00	0.73
Medium	265	0.829 (0.652, 1.055)		0.865 (0.670, 1.116)		13	0.539 (0.181, 1.609)		0.332 (0.092, 1.200)	
High	265	0.816 (0.642, 1.037)		0.877 (0.677, 1.135)		10	1.033 (0.322, 3.313)		0.876 (0.223, 3.445)	
log [MeFOSAA, ng/mL]	859	0.854 (0.695, 1.049)	0.13	0.929 (0.743, 1.161)	0.52	43	1.567 (0.601, 4.089)	0.36	1.414 (0.465, 4.293)	0.54

*OR* odds ratio, *CI* confidence interval

^a^ Based on menopausal status at blood draw; excludes 1 participant for whom menopausal status was unknown

^b^ Crude ORs adjusted for design variables of age at baseline enrollment, race/ethnicity, region of residence

^c^ For the categorical analysis, the *p*-values represent a test for linear trend with tertiles of PFAS modeled as a 3-level ordinal variable; for the continuous PFAS term, the *p*-value represents the *p*-value of the Wald-statistic for the β-coefficient for the PFAS modeled as a continuous term

^d^ For PFOA, PFNA, PFOS, MeFOSAA, covariates included in the fully-adjusted model were:

age at baseline enrollment, race/ethnicity, region of residence, blood draw date, blood draw date^2^, season of blood draw, total pack-years smoking, BMI, family history of breast cancer, age at first full-term pregnancy, pork consumption. For PFUnDA and PFHxS covariates included in adjusted models were: age at baseline enrollment, race/ethnicity, region of residence, blood draw date, blood draw date^2^, season of blood draw, total pack-years smoking, family history of breast cancer, age at first full-term pregnancy, pork consumption

^e^ For PFOA covariates included in all fully-adjusted models were: age at baseline enrollment (20–39/40–80+), race/ethnicity (white/non-white), region of residence, blood draw date, blood draw date^2^, season of blood draw, dietary fat, and total red meat consumption. For PFOS, PFUnDA, PFHxS, and MeFOSAA covariates included in all fully-adjusted models were: age at baseline enrollment (20–39/40–80+), race/ethnicity (white/non-white), region of residence, season of blood draw, and total red meat consumption. For PFNA covariates included in all fully-adjusted models were: age at baseline enrollment (20–39/40–80+), race/ethnicity (white/non-white), and region of residence

^f^ PFNA: Postmenopausal excludes *n* = 39 with non-reportable serum value, (cases: *n* = 30; controls: *n* = 9); Pre-/Peri-menopausal exclude *n* = 1 case with non-reportable serum value

When the data were stratified by tumor hormone responsiveness, no statistically-significant risks were observed for the 743 cases with ER+ or PR+ tumors (Table 5). However, among the 107 cases with tumors that were both ER- and PR-, adjusted ORs significantly below one were observed for PFUnDA and PFHxS. There was also some suggestion of an inverse association for PFOS among those with ER-/PR- tumors (*p* = 0.06). All other analyses conducted among the a priori selected subsets of interest yielded results similar to those observed in the full study population (*data not shown*).

**Table 5:** Invasive breast cancer risk associated with serum PFAS, by tumor hormone receptor status ^a^

PFAS Serum Concentration	ER+ or PR+ (*n* = 1601)					ER- and PR- (*n* = 965)				
	# cases	Crude Odds Ratio (OR) ^b^		Adjusted Odds Ratio (OR) ^d^		# cases	Crude Odds Ratio (OR) ^b^		Adjusted Odds Ratio (OR) ^e^	
		OR (95% CI)^b^	*p*-value^c^	OR (95% CI) ^d^	*p*-value^c^		OR (95% CI)^b^	*p*-value ^c^	OR (95% CI)^e^	*p*-value^c^
PFOA										
Low	266	1.00	0.50	1.00	0.71	43	1.00	0.38	1.00	0.39
Medium	247	0.923 (0.723, 1.178)		0.918 (0.707, 1.191)		35	0.824 (0.505, 1.346)		0.846 (0.510, 1.403)	
High	230	0.917 (0.711, 1.182)		0.952 (0.725, 1.251)		29	0.800 (0.474, 1.348)		0.792 (0.460, 1.365)	
log [PFOA, ng/mL]	743	0.781 (0.529, 1.151)	0.21	0.779 (0.513, 1.183)	0.24	107	0.523 (0.246, 1.111)	0.09	0.528 (0.239, 1.165)	0.11
PFNA										
Low	233	1.00	0.57	1.00	0.76	33	1.00	0.43	1.00	0.51
Medium	250	1.112 (0.868, 1.426)		1.034 (0.789, 1.354)		34	1.107 (0.659, 1.859)		1.002 (0.583, 1.720)	
High	234	1.076 (0.835, 1.387)		0.959 (0.724, 1.270)		36	1.231 (0.734, 2.064)		1.186 (0.690, 2.039)	
log [PFNA, ng/mL]	717 ^f^	1.096 (0.779, 1.543)	0.60	0.924 (0.634, 1.346)	0.68	103^f^	0.783 (0.416, 1.475)	0.45	0.701 (0.357, 1.375)	0.30
PFUnDA										
Low	249	1.00	0.38	1.00	0.22	43	1.00	0.03	1.00	0.05
Medium	274	1.116 (0.876, 1.422)		1.125 (0.866, 1.461)		41	0.947 (0.591, 1.518)		1.007 (0.614, 1.651)	
High	220	0.889 (0.689, 1.146)		0.838 (0.636, 1.106)		23	0.522 (0.298, 0.912)		0.550 (0.307, 0.985)	
log [PFUnDA, ng/mL]	743	0.922 (0.710, 1.196)	0.54	0.892 (0.671, 1.185)	0.43	107	0.661 (0.386, 1.133)	0.13	0.665 (0.375, 1.179)	0.16
PFHxS										
Low	287	1.00	0.10	1.00	0.25	54	1.00	0.02	1.00	0.03
Medium	219	0.763 (0.594, 0.980)		0.830 (0.635, 1.084)		28	0.582 (0.352, 0.962)		0.587 (0.350, 0.985)	
High	237	0.812 (0.629, 1.048)		0.855 (0.651, 1.122)		25	0.558 (0.326, 0.954)		0.567 (0.326, 0.985)	
log [PFHxS, ng/mL]	743	0.874 (0.640, 1.193)	0.40	0.915 (0.655, 1.279)	0.60	107	0.449 (0.250, 0.807)	0.01	0.446 (0.243, 0.821)	0.01
PFOS										
Low	250	1.00	0.83	1.00	0.81	47	1.00	0.09	1.00	0.06
Medium	247	1.020 (0.796, 1.305)		0.937 (0.721, 1.218)		32	0.697 (0.427, 1.139)		0.628 (0.378, 1.041)	
High	246	1.029 (0.798, 1.327)		0.967 (0.737, 1.270)		28	0.652 (0.387, 1.100)		0.615 (0.357, 1.059)	
log [PFOS, ng/mL]	743	1.177 (0.852, 1.626)	0.32	1.054 (0.744, 1.493)	0.77	107	0.645 (0.375, 1.109)	0.11	0.573 (0.323, 1.016)	0.06
MeFOSAA										
Low	289	1.00	0.08	1.00	0.23	36	1.00	0.91	1.00	0.89
Medium	228	0.802 (0.628, 1.023)		0.855 (0.660, 1.109)		39	1.106 (0.675, 1.814)		1.124 (0.674, 1.877)	
High	226	0.806 (0.630, 1.030)		0.854 (0.655, 1.113)		32	0.968 (0.575, 1.630)		0.960 (0.557, 1.654)	
log [MeFOSAA, ng/mL]	743	0.864 (0.699, 1.067)	0.18	0.932 (0.740, 1.173)	0.55	107	0.983 (0.631, 1.531)	0.94	0.996 (0.628, 1.578)	0.98

*OR* odds ratio, *CI* confidence interval, *ER+* Estrogen receptor positive, *PR+* Progesterone receptor positive, *ER-* Estrogen receptor negative, *PR-* Progesterone receptor negative

^a^ Excludes 52 cases with unknown tumor hormone receptor status

^b^ Models adjusted for age at baseline enrollment, race/ethnicity, region of residence

^c^ For the categorical analysis, the *p*-values represent a test for linear trend with tertiles of PFAS modeled as a 3-level ordinal variable; for the continuous PFAS term, the *p*-value represents the *p*-value of the Wald-statistic for the β-coefficient for the PFAS modeled as a continuous term

^d^ ORs for ER+ or PR+ cases adjusted for: age at baseline enrollment, race/ethnicity, region of residence, date of blood draw, date of blood draw^2^, season of blood draw, total smoking pack-years, BMI, family history of breast cancer, age at first full-term pregnancy, menopausal status at blood draw and pork consumption

^e^ ORs for ER- and PR- cases adjusted for (except PFNA): age at baseline enrollment, race/ethnicity, region of residence, date of blood draw, date of blood draw^2^, season of blood draw and physical activity. ORs for PFNA adjusted for: age at baseline enrollment, race/ethnicity, region of residence, date of blood draw, date of blood draw^2^ and season of blood draw

^f^ PFNA: ER+ or PR+ group excludes 26 cases with non-reportable serum values; ER- and PR- group excludes 4 with non-reportable serum values

## Discussion

Overall the findings from this study do not provide evidence that concentrations of PFASs measured in serum collected after diagnosis are related to breast cancer risk in this population of middle-aged and older California women. Our results are consistent with the null findings reported in a pair of C8 Science Panel Studies conducted among Ohio and West Virginia residents with known drinking water PFOA contamination [49, 50] and from a Danish population-based nested case-control study [51, 52] but stand in contrast to elevated risks reported from a small case-control study of Greenlandic Inuits [53–55]. Although both the Danish and Inuit study reported increased breast cancer risk associated with PFOSA [51], we could not evaluate risk for PFOSA due to the low frequency of detection in our study population. The inconsistency in findings across this small body of epidemiologic research may be a reflection of differing exposure profiles and host susceptibilities of the study populations considered, as well as features of study design.

The significantly elevated breast cancer risks found among the Greenlandic Inuits [53–55] may be a function of both their uniquely high PFAS exposures and potentially greater genetic susceptibility to risks associated with those exposures. The serum levels of some PFASs reported in the Inuits are especially high compared to the levels in our population, as well as those reported in other areas of the world [68]. Specifically, in comparison to the median levels in our controls, the median levels in the Inuit controls were approximately 162%, 200%, and 1700% higher for PFOS, PFNA, and PFUnDA, respectively (Additional file 1: Table S1). Thus the elevated breast cancer risks observed for PFOS among the Inuits but not in our study may be a reflection of their higher exposures. However, for PFOA and PFHxS (two PFASs for which elevated breast cancer risks were observed in the Inuits and not in our study) the median levels in the Inuits were approximately 59 and 67% lower (respectively) than in our study. Furthermore, PFOA concentrations in the Danish study, and particularly the C8 Science Panel Study, both of which reported no breast cancer risks, were much higher than in the Inuit. Thus, it seems unlikely that the positive findings among the Inuits are solely a function of high PFAS exposures. This Inuit population, however, also has notably high body burdens of a number of other persistent organic pollutants (POPs) that have been suspected to play a role in breast cancer risk, including polychlorinated biphenyl ethers (PCBs) [69] of which some are much higher than in our study population. It is possible that uncontrolled confounding for exposures to other carcinogenic or xenoestrogenic compounds such as PCBs could be driving the positive PFAS effects reported in the Inuits. In fact, increased risks of breast cancer were also reported in the Inuit study for a number of PCBs [55].

The increased breast cancer risks observed among the Greenlandic Inuits may also reflect greater susceptibility to PFAS exposures. Analyses of genetic polymorphisms in genes that are involved in estrogen metabolism and estrogen biosynthesis (e.g. CYP, COMT) indicated polymorphic frequencies in this Inuit population that are distinctly different than those reported in Euro-Caucasian populations that have been the focus of the other breast cancer studies to date [54]. While the Inuit study was under-powered to statistically evaluate gene-environment interactions, there was some indication that the risks associated with PFASs and other POPs were more pronounced in those with specific polymorphisms in the targeted genes of interest [54]. This is an area of inquiry that deserves additional attention.

Another potential explanation for the disparate findings across the small body of studies conducted to date could be the variability in methodologic approaches, especially with regards to exposure assessment. Our approach for estimating PFAS exposure was more similar to that used in the Greenlandic Inuit study but our findings are more consistent with the null results reported from the Danish [51, 52] and C8 Science Panel [49, 50] studies which used markedly different exposure ascertainment methods. Both our study and the Inuit study relied on serum concentrations of PFAS measured in blood that was collected after diagnosis. While single measures of PFAS in serum are generally regarded as reasonable measures of chronic exposures [68], the degree to which PFAS concentrations may be affected by hormonal or other physiologic changes associated with the onset of and/or sequelae and treatment of breast cancer is not known. Thus, the possibility of reverse causation bias in our study and the Inuit study cannot be fully discounted.

An important distinction between our study and the Inuit study is that while both relied on blood collected post-diagnosis, in the Inuit study the blood was collected prior to treatment while in our study this was not the case. To our knowledge, the effects of breast cancer treatment, if any, on levels of serum PFAS have not been explored and remain unknown. If breast cancer treatment causes declines in PFAS levels, this would have limited our ability to detect an increase in breast cancer risk and could have resulted in spurious inverse associations. Unfortunately, complete information on treatment is not available for the CTS cohort.

Exposure assessment in the C8 Science Panel Study, while retrospective, was not based on body burden measurements but rather was derived from complex modeling incorporating information from self-reported residential histories, historical data on PFOA drinking water contamination, occupational histories and a job exposure matrix to estimate cumulative PFOA exposures. While these estimates were validated with serum PFOA levels in a subset of participants, the possibility of exposure misclassification remains.

A unique strength of the Danish study is its reliance on quantitatively-measured values of PFASs in blood samples collected 10 to 15 years prior to breast cancer diagnosis/enrollment. Notably, however, the blood samples, were collected during early pregnancy. This poses some complication in the interpretation of the study’s results. Pregnancy-related changes in blood volume and alterations in concentrations of serum albumin [70] to which PFASs bind, may affect PFAS serum levels. Analyses of NHANEs data have suggested lower PFOS levels in pregnant women [71]. Furthermore, the expulsion of the fetus and placenta and blood loss accompanying parturition is considered a major route of PFAS elimination among parous women [72]. Thus, the measurement of PFASs in blood specimens collected during pregnancy may not be a valid representation of chronic PFAS exposures and the null results reported for most of the PFAS examined by the Danish study should not be regarded as evidence that chronic exposures are not related to breast cancer risk. On the other hand, pregnancy is considered an important window of susceptibility during which breast tissue may be particularly vulnerable to the deleterious effects of endocrine disrupting and carcinogenic compounds [73]. Findings from the Danish study suggest that other than possibly for PFOSA, PFAS exposures during this time do not impact breast cancer risk.

Overall we interpret the results from our study as null. Our analyses, however, indicated some suggestion of an inverse association between breast cancer risk and some PFASs, specifically PFUnDA and PFHxS, that appeared to be driven by risks among cases with non-hormonally responsive tumors. We find these results to be particularly perplexing. Given the body of literature on the potential estrogen disrupting properties of these compounds (with some studies suggesting anti-estrogenic properties), the most logical explanation for these findings would be that they are a reflection of anti-estrogenic activity. If this were the case, we would expect these effects to be more pronounced among cases with hormonally responsive tumors, as the etiology of these tumors is thought to be more strongly driven by hormonal factors than tumors that are not hormonally responsive [74, 75]. We, in fact, found just the opposite, with more robust and pronounced inverse associations among the cases with hormonally non-responsive tumors and no statistically significant effects among the cases with ER+/PR+ tumors. Given the small number of cases upon which these estimates are based and the lack of a plausible biologic mechanism (at least based on our current state of knowledge), we regard these findings as either due to chance, or to be an artifact of our study design. In particular, the measurement of PFAS after treatment, coupled with our inability to account for treatment may be relevant.

To our knowledge, it is not known whether breast cancer treatment affects serum PFAS levels but it is conceivable that certain treatments, such as chemotherapy and hormonal therapy, could alter PFAS levels. One small study that compared serum levels of chlorinated hydrocarbons in breast cancer patients pre- and post-treatment reported significant differences in levels among patients who had received chemotherapy [76]. It is difficult to ascertain whether such findings are relevant to PFASs, which in contrast to the chlorinated hydrocarbons, are not lipophilic and would likely not be influenced by changes in body weight that could be associated with certain breast cancer treatments. The recommended course of treatment does differ for women with hormonally responsive and non-responsive tumors. Thus, it is plausible that the suggestive protective effects we observed for PFUnDA and PFHxS that appear to be confined to cases with ER-/PR- tumors could be a spurious artifact of the recommended treatment regimens more commonly prescribed for these breast cancer patients.

However, these suggestively inverse findings should not be entirely dismissed. A significant inverse association between breast cancer risk and PFHxS was also reported by the Danish study (RR = 0.66, 95% CI = 0.47–0.94 for logged serum concentrations) [51] and for PFOA in one of the C8 Science Panel studies (HR = 0.93, 95%CI = 0.88–0.99 for a logged estimated 10-year lagged cumulative PFOA serum concentration) [49]. Furthermore, these findings are consistent with evidence, although limited, from epidemiologic and animal studies that suggest PFAS exposures may delay the onset of puberty [30, 32, 67] and lead to earlier menopause [77], both of which are conditions associated with reduced risks of breast cancer. In our study, neither age at menarche nor age at menopause were identified as significant confounders in our risk models and risks did not substantially differ when we stratified our analyses by categories of age at menarche or menopause (*data not shown*). Moreover, age at menarche did not correlate with serum PFAS levels *(data not shown*). A significant but very weak inverse correlation (*r* < 0.10), however, was seen between age at menopause and serum level of PFHxS but not PFUnDA (*data not shown*). Because the serum PFAS levels in our study were measured after menarche and menopause (in post-menopausal women), it is not possible to directly assess whether the suggestively protective effects we observed for some of the PFASs could be mediated by effects on the timing of menarcheal and menopausal onset.

When interpreting the results of our study it is important to consider potential mechanistic pathways. Three pathways have been proposed by which environmental chemicals such as the PFASs can impact breast cancer risk: 1) developmental toxicity mediated by endocrine disruption, leading to permanent morphologic changes in breast tissue and/or alterations in sex-steroid hormonal signaling pathways; 2) genotoxicity, either direct or indirect; and 3.) hormonal tumor promotion. Within each of these pathways, dose and timing of exposure are critical factors in determining effects [78].

For the PFASs, evidence is most compelling from both laboratory and human studies for developmental toxicity associated with exposures encountered during fetal through pre/peri-pubertal development [4, 30, 32, 67]. Our study, however, may not be particularly well-suited to evaluate breast cancer risks mediated through this pathway, at least for some of the PFASs, for which human exposures may not have become widespread until after our study subjects had passed through these important developmental windows of susceptibility. The first PFASs were introduced in the early 1950s. All the women in our study were born before 1972 and most (approximately 75%) were born 1950 or earlier. Thus, the age structure of our study population makes it unlikely that many of our study participants would have been exposed to PFAS during fetal development. A lack of population-based historical biomonitoring data prior to the 1990s makes it difficult to ascertain how much opportunity for exposure our study participants would have had during the pre/peri-pubertal stages of development. While widespread human exposures first became widely recognized in the 1990s (thanks to the emergence of population-based biomonitoring programs), there is not sufficient historical biomonitoring data to clearly establish when human exposures became prevalent in the U.S. A recent temporal analysis of serum PFAS concentrations in three groups of California women collected in the 1960s, 1980s and 2009, however, suggests that elevated population exposures for at least some PFASs (specifically PFOS and PFHxS) may have been widespread as far back as the 1960s while widespread elevated exposures to other PFASs, particularly the longer chain perfluorocarboxylic acids such as PFOA and PFNA, may not have emerged until the 1980s or later [59].

Genotoxic effects are initiated through genetic damage which may lead to mutations that eventually progress to cancer. Mammary tissue appears to be most susceptible to the genotoxic effects of carcinogens during puberty and pregnancy when the mammary cells are not fully differentiated and are undergoing rapid proliferation [73]. However, exposures encountered at any time have the potential for carcinogenic genotoxicity. Although still a topic of some controversy, the current consensus based on evidence from laboratory data is that PFASs are not directly genotoxic but may be indirectly genotoxic through the formation of reactive oxygen species in response to oxidative stress [36, 39, 69, 78].

The mechanistic pathway for which our study is perhaps best suited to provide insight is that of hormonally-mediated tumor promotion. Many of the primary risk factors for breast cancer are thought to be mediated by cumulative lifetime exposure to high levels of endogenous estrogens [79] and the recent recognition of increased breast cancer risks associated with the use of menopausal hormone therapy [80] further highlights the potential impact of exposures to exogenous estrogens encountered later in life on breast cancer risk.

The estrogen disrupting properties of PFASs remain unclear. A fairly large body of laboratory studies, and to a lesser extent human studies, suggest myriad and complex potential estrogen disrupting effects which are far from well-understood. In laboratory studies, PFASs have been shown to both increase and decrease estradiol levels [4, 66, 67], with effects varying by species (and strains within species) [4, 32], by PFAS concentrations (with a dose-response relationship that is not necessarily monotonic) [66], and by specific PFAS [4, 51, 66]. Moreover, epidemiologic and in vitro data further suggest that the estrogen disrupting properties of PFASs may be highly dependent on co-exposure levels of endogenous estradiol. For example, PFOA exposures have been shown to be estrogenic in isolation but anti-estrogenic with co-exposure to high levels of estradiol [66, 67, 81]. Furthermore, recent epidemiologic data suggest that some PFASs may only act as estrogen disruptors among nulliparous women [72, 82]. For example, in a study of naturally cycling premenopausal women, Barrett and colleagues reported reductions in circulating estradiol associated with increases in serum PFOS concentrations but only among nulliparous, not parous women [72].

While we conducted a number of analyses among subsets of our study population designed to crudely capture groups of women who might have distinctly higher or lower levels of circulating endogenous estrogens (e.g. stratification by menopausal status, parity, categories of BMI, never/ever users of menopausal hormone therapy), these analyses proved to be non-illustrative. Without the ability to control for endogenous estrogen levels, both our generally null findings, and suggestively protective findings for some PFASs, may be an artifact of an inability to account for endogenous estrogen levels which could impact the estrogenic or anti-estrogenic effects of PFAS, if they exist.

The suggestion of a potentially protective effect associated with a few of the PFASs however warrants further attention, especially in light of toxicological evidence suggesting potential anti-estrogenic properties for some of these compounds under certain conditions. In particular, it appears that endogenous estrogen levels may be especially important in determining the mode of action of PFASs, including whether they exert estrogenic or anti-estrogenic properties. Future studies of this issue that can incorporate information about endogenous estrogen levels or genetic determinants of estrogen biosynthesis and metabolism would be particularly informative.

In summary, it is important to consider our findings in the context of the strengths and limitations of our study. The age structure of our study population limited our ability to evaluate risks associated with in utero exposures, which may be the most etiologically relevant time period for breast cancer risk. Furthermore, the collection of blood samples post-diagnosis and post-treatment calls into question the possibility of reverse causality. As with any epidemiologic case control study, the potential for selection bias and uncontrolled confounding also must be considered. However, our reliance on data collected from a case control study nested within an on-going cohort study that was specifically designed to study breast cancer, somewhat minimizes these concerns. The eligibility criteria for our study population was well-specified, cases and controls were carefully matched, and we saw little evidence of survival bias. The wealth of data collected on known breast cancer risk factors allowed for the evaluation of a comprehensive set of potential confounders.

## Conclusions

The results from this study do not support an association between serum levels of PFASs and risk of breast cancer. Interpretation of these results, however, should not be overextended to conclude that PFASs are not of concern for breast cancer. In the context of the large body of laboratory data demonstrating endocrine disrupting properties of these PFASs at concentrations similar to those seen in some human populations, further study is warranted. It is especially important to elucidate mechanisms of action. Exposure to many of the PFASs examined in our study are declining due to regulatory and voluntary phase-outs enacted in response to heath concerns. These compounds however are being replaced with other PFASs that have not yet been studied [2, 23, 83]. A greater mechanistic understanding of how, and if, the PFASs influence breast cancer and other health outcomes is critical to avoiding “regrettable substitutions” of these compounds with replacement chemicals that may have similar toxicological properties and health consequences.

## Additional file

Additional file 1: **Table S1.** Comparison of serum PFAS levels (ng/mL) in this study with levels in other epidemiologic studies of breast cancer risk. (DOCX 25 kb)

## Abbreviations

BMI: Body mass index
CCR: California Cancer Registry
CI: Confidence interval
CTS: California Teachers Study
ER-: Estrogen receptor negative
ER+: Estrogen receptor positive
EtFOSSA: 2-(N-Ethyl-perfluorooctane sulfonamido) acetic acid
HT: Hormone therapy
MeFOSAA: 2-(N-Methyl-perfluorooctane sulfonamido) acetic acid
OR: Odds ratio
PFAS: Per- and poly- fluoroalkyl substances
PFBS: Perfluorobutane sulfonic acid
PFDA: Perfluorodecanoic acid
PFDoDA: Perfluorododeconic acid
PFHpA: Perfluoroheptanoic acid
PFHxS: Perfluorohexane sulfonic acid
PFNA: Perfluorononanoic acid
PFOA: Perfluorooctanoic acid
PFOS: Perfluorooctane sulfonic acid
PFOSA: Perfluorooctane sulfonamide
PFUnDA: Perfluoroundecanoic acid
PR-: Progesterone receptor negative
PR+: Progesterone receptor positive
US EPA: United States Environmental Protection Agency
