# Supplementary material for: Breast cancer risk and serum levels of per- and poly-fluoroalkyl substances: a case-control study nested in the California Teachers Study
Source: Environ Health. 2018 Nov 27;17:83. doi: 10.1186/s12940-018-0426-6 (PMC6260688; doi:10.1186/s12940-018-0426-6)
Supplement: Supplementary file 1 — Table S1. Comparison of serum PFAS levels (ng/mL) in this study with levels in other epidemiologic studies of breast cancer risk. (DOCX 25 kb) [file 12940_2018_426_MOESM1_ESM.docx]

**Additional file 1**

| **Study** | **Metric/**  **(Method, years)** | **PFOA** | **PFOS** | **PFNA** | **PFUnDA** | **PFHxS** |
| --- | --- | --- | --- | --- | --- | --- |
| Our Study | Median in Controls  (Measured in Sera, 2011-2015) | 2.48 | 6.95 | 0.85 | 0.13 | 1.61 |
| Greenlandic Inuit  (Wielsoe et al. 2017) | Median in Controls  (Measured in Sera, 2000-2003)  Median in Controls  (Measured in Sera, 2011-2014) | 1.62  1.45 | 18.1  18.2 | 0.89  2.58 | 0.98  2.35 | 1.38  1.08 |
| C8 Science Panel Study  (Barry et al. 2013) | Median in community cohort  (Modeled, 1952-2011) | 24.2 |  |  |  |  |
| Our Study | Means in Controls  (Measured in Sera, 2011-2015) | 2.94 | 8.32 | 1.04 | 0.16 | 2.24 |
| Danish Premenopausal  (Bonefeld-Jorgensen et al. 2014) | Means in Controls  (Measured in Sera, 2000-2003) | 5.2 | 30.96 | 0.5 | -- | 1.2 |

**Table S1. Comparison of serum PFAS levels (ng/mL) in this study with levels in other epidemiologic studies of breast cancer risk.**
